# Definition und Darstellung der verschiedenen Typen des Nasenzyklus mittels Langzeitrhinometrie

**DOI:** 10.1007/s00106-021-01078-1

**Published:** 2021-06-25

**Authors:** E. F. Reins, C. Weindel, T. K. Hoffmann, F. Sommer, F. Stupp, A.-S. Halbig, J. Lindemann

**Affiliations:** 1 Klinik für Hals‑, Nasen- und Ohrenheilkunde, Kopf- und Halschirurgie, Universitätsklinikum Ulm, Frauensteige 12, 89075 Ulm, Deutschland; 2 Universität Ulm, Albert-Einstein-Allee 11, 89081 Ulm, Deutschland

**Keywords:** Nasenzyklus, Langzeitrhinometrie, Nasenschleimhaut, Rhinomanometrie, Operationsplanung, Nasal cycle, Long-term rhinometry, Nasal mucosa, Rhinomanometry, Surggery planning

## Abstract

**Hintergrund:**

Als „klassischer Nasenzyklus“ gilt ein streng wechselseitiges An- und Abschwellen der Mukosa der linken und rechten Nasenseite. Erfahrungen aus dem klinischen Alltag und Berichte in der Literatur lassen jedoch auf ein komplexeres Bild schließen. Die Erkenntnisse über den Nasenzyklus beruhten bisher auf Einzelmessungen. Die Langzeitrhinometrie (LRM) ermöglicht nun erstmals eine kontinuierliche Untersuchung des nasalen Luftflusses. Ziel dieser Untersuchung war daher die Evaluation des Nasenzyklus mit der LRM in einem nasengesunden Probandenkollektiv über 24 h.

**Methodik:**

Bei 55 rhinologisch gesunden Probanden wurde eine LRM mit dem tragbaren Messsystem Rhino-Move© (Happersberger Otopront, Hohenstein) über 24 h durchgeführt.

**Ergebnisse:**

Neben der erwarteten streng wechselseitigen Schwellung der Mukosa im Sinne des „klassischen Nasenzyklus“ zeigten sich weitere wiederkehrende Zyklustypen: der „in-concert“ Typ mit simultaner Fluktuation des Luftstromes auf beiden Nasenseiten, der „einseitige Typ“ mit An- und Abschwellen der Schleimhaut auf nur einer Nasenseite und der „non-cycle“ Typ ohne darstellbare Veränderung des Luftstromes auf beiden Seiten. Die meisten Probanden zeigten innerhalb der 24 h eine Kombination verschiedener Zyklustypen („gemischter Typ“), die sich häufig bei Tag und Nacht unterschieden.

**Diskussion:**

Unsere Untersuchung bestätigt die Vermutung, dass der Nasenzyklus gemessen über 24 h wesentlich komplexer ist als in der Literatur beschrieben. Die meisten Probanden zeigen mehrere der hier beschriebenen 5 Zyklustypen. Die LRM hat sich dabei als leicht zu bedienende und zuverlässige Messmethode erwiesen. Der Zusammenhang zwischen Zyklustyp und körperlicher Aktivität sowie anderen Faktoren bleibt zu erforschen.

## Abstract (en)

**Background:**

A reciprocal swelling of the nasal mucosa is often referred to as the classical nasal cycle; however, reports in the literature suggest a more complex picture. Most of the research on the nasal cycle is based on individual measurements. The long-term rhinometry (LRM) now makes it possible to continuously examine the cyclic swelling of the nasal mucosa over 24 h. The aim of this study was therefore to evaluate the nasal cycle with LRM over 24 h.

**Material and methods:**

An LRM was performed in 55 rhinologically healthy subjects over 24 h using the portable measuring system Rhino-Move© (Happersberger Otopront; Hohenstein, Germany).

**Results:**

In addition to the expected strictly reciprocal swelling of the nasal mucosa in the sense of the classical nasal cycle, the following cycle types were detected: in-concert type with simultaneous rise and drop of the air flow on both sides of the nose, the one-sided type with significant congestion and decongestion of the mucous membrane only on one side and no detectable changes on the other side of the nose and the non-cycle type without any change in airflow on both sides. Most subjects showed a complex picture with multiple cycle types within the 24 h measurement (mixed nasal cycle). The types often differed during the day and night.

**Conclusion:**

This study confirms the assumption that the nasal cycle measured over 24 h is much more complex than often described in the literature. Most subjects showed several of the 5 cycle types described here. The LRM has proven to be an easy to- use and reliable measurement method. The relationship between cycle type and physical activity as well as other factors remains to be investigated.

Als „klassischer Nasenzyklus“ gilt ein streng wechselseitiges Anschwellen der nasalen Mukosa. Regulation und Funktion des Nasenzyklus sind bis heute nicht vollständig verstanden, vielmehr scheint er wesentlich komplexer zu sein als bislang angenommen. Die Untersuchung des Nasenzyklus basierte bisher hauptsächlich auf Einzelmessungen über wenige Stunden am Tag. Die Langzeitrhinometrie (LRM) ermöglicht nun erstmals die kontinuierliche Messung des nasalen Luftflusses. In diesem Beitrag schlagen wir anhand unserer Ergebnisse der LRM, gemessen über 24 Stunden, die Einteilung des Nasenzyklus in 5 Typen vor.

## Hintergrund

Als Nasenzyklus wird das zyklische und auf beiden Seiten alternierende An- und Abschwellen der nasalen Mukosa bezeichnet [17, 18]. Das Phänomen ist bisher noch nicht abschließend untersucht und verstanden. Nach heutigem Kenntnisstand scheint der Nasenzyklus jedoch an wichtigen respiratorischen und immunologischen Aufgaben der Nase beteiligt zu sein [5]. Über das autonome Nervensystem gesteuert führt der variierende Füllungszustand des erektilen Gewebes aus venösen Sinusoiden und Plexus in den Nasenmuscheln sowie am anterioren Septum zu konstanter Atemluftmenge und -befeuchtung [4, 11]. Als übergeordneter Taktgeber wird der Hypothalamus angenommen [31].

In der Vergangenheit erfolgte eine Reihe von Studien mit unterschiedlichen Messmethoden, um das Phänomen des Nasenzyklus zu untersuchen. Neben der aktiven, anterioren Rhinomanometrie wurden im Verlauf die Rhinoresistometrie [21, 30] und die akustische Rhinometrie sowie eine Kombination dieser Methoden [7, 8, 10, 14] tagsüber eingesetzt. Zusätzlich wurden Untersuchungen mittels PNIF („peak nasal inspiratory flow“), MRT (Magnetresonanztomographie) und numerischen Simulationen durchgeführt [23]. Diese in der Klinik durchaus gängigen Untersuchungsmethoden ermöglichen jedoch jeweils nur eine Momentaufnahme, und alltägliche Aktivitäten müssen zwangsweise unterbrochen werden. Eine kontinuierliche Untersuchung des Schwellungsverhaltens der nasalen Mukosa während Phasen des Schlafes oder von starker körperlicher Aktivität ist so nicht möglich.

Bereits 1895 beschrieb Richard Kayser erstmalig die wechselnde Luftdurchlässigkeit der beiden Nasenseiten, die er mithilfe einer Röhrenvorrichtung über den Mund ableitete [15]. Der Großteil der Studien, die zur Erforschung des Nasenzyklus durchgeführt wurden, untersuchte seither ein Phänomen, bei dem es streng wechselseitig zum Anschwellen der Schleimhaut auf einer Nasenseite kommt, während die andere Seite abgeschwollen ist und „ruht“ [12]. Dieser „klassische Nasenzyklus“ konnte in mehreren Studien bei etwa 80 % der erwachsenen Bevölkerung nachgewiesen werden [13, 26, 27]. Einzelne Studien lassen jedoch erahnen, dass sich das Schwellungsverhalten der nasalen Mukosa weitaus komplexer darstellt und es neben dem „klassischen Nasenzyklus“ noch weitere Zyklustypen gibt [13, 16]. Bislang gibt es aber keine einheitliche Einteilung der verschiedenen Zyklustypen.

In der Erforschung des nasalen Schwellungsverhaltens entwickelte die Arbeitsgruppe um Prof. Dr. G. Mlynski die sogenannte Langzeitrhinometrie (LRM; [9]). Mit dieser Entwicklung steht nun erstmals eine Untersuchungsmethode zur Verfügung, die eine kontinuierliche Aufzeichnung des nasalen Luftstroms über 24 h und damit eine lückenlose Untersuchung des Nasenzyklus auch bei körperlicher Anstrengung und im Schlaf ermöglicht [26]. Hierzu gibt es bisher nur einzelne Publikationen [2, 9, 22, 26], ferner findet sich bisher keine einheitliche Beschreibung oder Systematik der verschiedene Zyklustypen über 24 h.

Das Ziel dieser Untersuchung war daher die Beobachtung und Beschreibung der zyklischen Veränderungen der Nasenschleimhaut in einem größeren nasengesunden Probandenkollektiv über 24 h und eine Definition verschiedener Nasenzyklustypen.

## Material und Methoden

Die Studie wurde durch die Ethikkommission der Universität Ulm genehmigt.

### Probanden

Wir führten bei 55 nasengesunden Probanden (36 Frauen, 19 Männer) im Alter zwischen 19 und 80 Jahren (mittleres Alter 37,4 Jahre ±18,2) eine LRM über 24 h durch. Die Auswahl der Probanden erfolgte anhand eines Fragebogens, durch den nasale Symptome, wie eine permanente Nasenatmungsbehinderung und Allergien mit nasaler Beteiligung sowie die Einnahme von Medikamenten mit Auswirkungen auf die Schleimhäute (z. B. ACE[„angiotensin converting enzyme“]-Hemmer, Antidepressiva mit anticholinerger Wirkung, Antihistaminika, Nasensprays), ausgeschlossen wurden. Ausgeschlossen wurden zudem Raucher, Schwangere sowie Probanden mit chronischem bronchialen Asthma, Traumata oder Operationen der Nase in der Anamnese. Durch die anschließende anteriore Rhinoskopie und nasale Endoskopie wurden strukturelle Auffälligkeiten der äußeren und inneren Nase sowie nasale Polypen ausgeschlossen. Ein Pricktest war bei allen eingeschlossenen Probanden negativ.

### Messsystem

Das tragbare Messsystem „Rhino-Move©“ (Happersberger Otopront GmbH, Hohenstein) zeichnet den nasalen Luftstrom seitengetrennt mithilfe einer speziellen Nasenbrille konstant über 24 h als Differenz von dynamischem und statischem Druck auf. Zusätzlich werden die Atemfrequenz und, über ein 3‑Kanal-Elektrokardiogramm (EKG), die Herzfrequenz aufgezeichnet.

Nach Anlegen und Kalibrierung des Messsystems mithilfe des Rhinomanometers der Rhino-Sys-Einheit (Happersberger Otopront GmbH, Hohenstein) erfolgte eine Einweisung in die Handhabung und der Start der Aufnahme. Die Probanden wurden angewiesen, das Gerät bei allen alltäglichen Tätigkeiten und in der Nacht zu tragen.

### Auswertung der Langzeitrhinometrie

**Figure 1:**
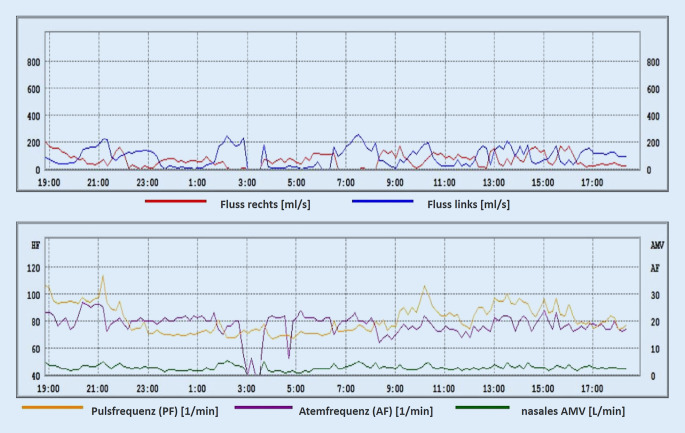

Die zum Rhinometriesystem „Rhino Sys“ gehörende Software (Version 1.8.2, Happersberger Otopront GmbH, Hohenstein) stellt die ermittelten Fluss- und Pulswerte in Form von 2 Liniendiagrammen (Abb. 1) dar. Das erste Diagramm zeigt die maximalen inspiratorischen Flusswerte seitengetrennt und farblich kodiert in ml/s, wobei jeweils die Mittelwerte aus 10-minütigen Intervallen bestimmt werden. Im zweiten Diagramm werden Pulsfrequenz (1/min), Atemfrequenz (1/min) sowie das gesamten nasale Atemminutenvolumens (l/min) aufgezeigt. Vor allem aus dem ersten Diagramm lässt sich so über den Zeitraum von 24 h bereits ein bestimmtes Muster des Schwellungsverhaltens der beiden Nasenseiten ableiten.

Die Flussdiagramme wurden ausgewertet und in Anlehnung an die Beschreibungen von Kern [17], Fisher et al. [6] und Mlynski [19] wurde eine Systematik von Zyklustypen erstellt und definiert.

## Ergebnisse

### Schema der Zyklustypen

**Figure 2:**
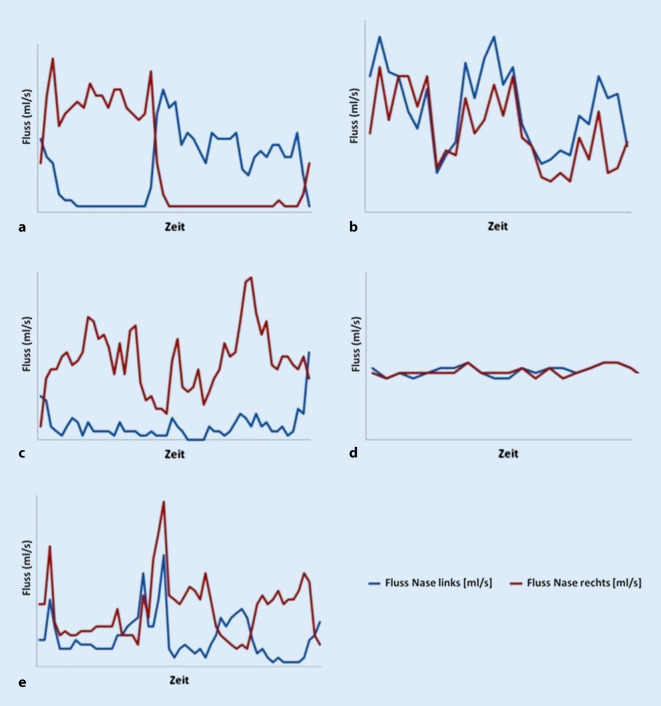

Nach Auswertung der Flussdiagramme aus 55 LRM(Langzeitrhinometrie)-Messungen konnten wir die folgenden wiederkehrenden Muster feststellen und schlagen daher die anschließende Einteilung in 5 Nasenzyklustypen vor:

- *„Klassischer“ Typ*: Es kommt zu einem reziproken Wechsel von An- und Abschwellen der Schleimhaut beider Nasenseiten. Die sich in der Arbeitsphase befindliche Seite zeichnet sich dabei durch eine hohe Flussgeschwindigkeit aus, während sich die andere Seite in der Ruhephase befindet. Beginn und Ende einer Phase kann durch die Schnittpunkte der Kurven des nasalen Blutflusses links (*blau*) und rechts (*rot*) in Abb. 2a definiert werden.
- *„In-concert“ Typ*: Hier zeigt sich ein simultaner Rhythmus aus Anstieg und Abfall des Luftstromes in beiden Nasenseiten (Abb. 2b).
- *Einseitiger Typ*: Ein signifikantes An- und Abschwellen der Schleimhaut kann nur auf einer Nasenseite detektiert werden, während es auf der Gegenseite zu keinem wesentlichen Wechsel zwischen Arbeits- und Ruhephase der Schleimhaut kommt (Abb. 2c).
- *„Non-cycle“ Typ*: Durch die herkömmlichen Messungen ist hier auf beiden Nasenseiten keine Veränderung des Luftstromes darstellbar. (Abb. 2d)
- *Gemischter Nasenzyklus:* Im Verlauf der 24-stündigen Messung treten mehrere der oben genannten Zyklusformen zu verschiedenen Zeiten tags und nachts auf (Abb. 2e).

#### Beispiele der Zyklustypen

**Figure 3:**
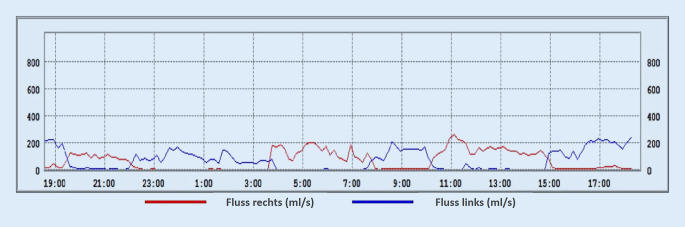

**Figure 4:**
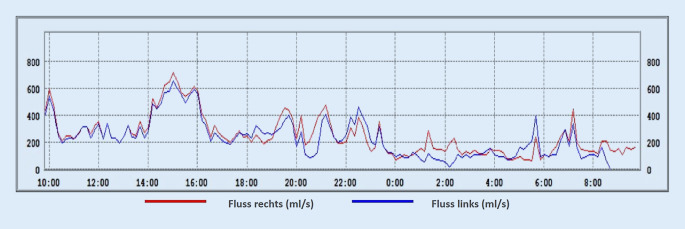

**Figure 5:**
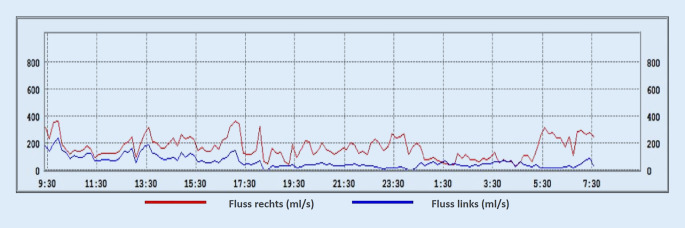

**Figure 6:**
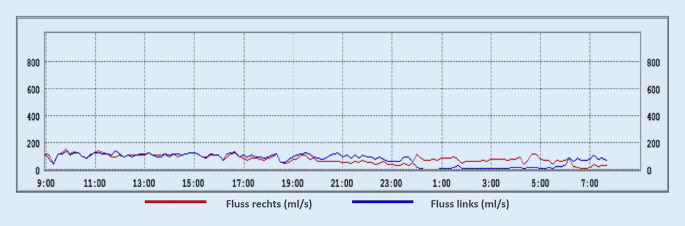

Im Folgenden präsentieren wir einige repräsentative Beispiele der in den LRM-Befunden gefundenen Zyklustypen anhand von Diagrammen, die den nasalen Luftfluss über die Zeit zeigen. Den LRM-Befund eines 24 Jahre alten männlichen Probanden, dessen nasaler Luftfluss sich über die gesamte Messdauer von 24 h im klassischen Sinne auf beiden Seiten abwechselt, zeigt Abb. 3. Im Gegensatz dazu weist der 25 Jahre alte männliche Proband in Abb. 4 einen reinen „in-concert“ Typen über 24 h auf. Es fällt auf, dass der Nasenzyklus der meisten Probanden, über 24 h gemessen, mehrere verschiedene der oben genannten Zyklustypen (Abb. 2a–d) aufwies („gemischter Typ“, Abb. 2e). So besteht der LRM-Befund des männlichen, 55 Jahre alten Probanden aus Abb. 5 aus „in-concert“ und „einseitigen“ Anteilen, während Abb. 6 den Nasenzyklus eines männlichen 75 Jahre alten Probanden mit „non-cycle“ und „klassischen“ Anteilen zeigt.

## Diskussion

Das wechselseitige An- und Abschwellen der Nasenschleimhaut der linken und rechten Seite an den Nasenmuscheln sowie am Septum wird in der Literatur als „klassischer“ Nasenzyklus bezeichnet [12]. Einzelne Studien lassen jedoch eine komplexere Systematik hinter dem Schwellungsmuster der nasalen Schleimhaut erahnen. Bislang gibt es keine einheitliche Einteilung des Nasenzyklus in verschiedene definierte Typen. Ziel dieser Arbeit war es daher, die zyklischen Veränderungen des Schwellungsverhaltens der nasalen Mukosa anhand eines größeren Probandenkollektivs (*n* = 55) kontinuierlich über 24 h mit der neuen Messmethode der LRM zu beschreiben und die beobachteten Zyklustypen zu definieren.

Bei jedem der 55 nasengesunden Probanden konnte während der Messzeit von 24 h mit der LRM mindestens einer der vorgestellten Nasenzyklustypen detektiert werden. Es fällt auf, dass die allermeisten Probanden sogar mehrere Zyklustypen und damit einen gemischten Nasenzyklus in 24 h aufweisen.

### Die „non-cycle nose“

Ausgehend von der Definition, dass ein Nasenzyklus vorliegt, sobald ein wechselseitiges An- und Abschwellen der Nasenschleimhaut nachweisbar ist, veröffentlichte E. Kern 1981 seine Arbeit „The noncylce nose“ [17]. Bei 14 von 50 Probanden konnte durch eine Rhinomanometrie kein klassischer Nasenzyklus nachgewiesen werden. Aus den Beobachtungen dieser Probanden definierte Kern 3 Typen der „non-cycle nose“: Typ I ohne jegliche Nachweisbarkeit einer Widerstandsveränderung auf beiden Seiten (8/14 Probanden), Typ II mit nachweisbaren Fluktuationen nur auf einer Nasenseite, während der Widerstand auf der anderen Seite konstant blieb (2/14) und Typ III mit simultanen Fluktuationen auf beiden Seiten („in-concert“) ohne erkennbare Dominanz (4/14; [17]). Aus seinen Beschreibungen lässt sich auch bei einem Teil der Probanden ohne nachweisbaren „klassischen Nasenzyklus“ eine gewisse Rhythmik erahnen. Jedoch wurden von Kern nur wiederholte Einzelmessungen über mehrere Stunden durchgeführt. Eine vollständige Aufzeichnung der Flow-Werte über 24 h erfolgte also nicht. Auch die Gruppe um G. Mlynski schlug anhand ihrer Erfahrungen mit der LRM bereits eine – im Gegensatz zur Konzentration auf den klassischen Nasenzyklus – komplexere Systematik, in ihrer Operationslehre vor [19].

### Die verschiedenen Zyklustypen

Der „klassische“ Nasenzyklus scheint ein wichtiger Mechanismus für die Funktionen der Nase zu sein. Es wird angenommen, dass der regelmäßige Wechsel zwischen An- und Abschwellen der Schleimhaut einen Gradienten zwischen Luft und Schleimhaut aufbaut, der die Abgabe von Wärme und Feuchtigkeit an die Atemluft bei der Inspiration ermöglicht [16, 20]. Für die Regulation der Atemluftmenge und für die Wahrnehmung des Luftstromes ist ein enger Kontakt zwischen Luft und Schleimhaut erforderlich. Die rhythmische Verschiebung der Phasen um 180° ermöglicht einen konstanten mittleren Atemluftstrom in der gesamten Nase [8, 28].

Hinter dem gleichzeitigen An- und Abschwellen der Nasenschleimhaut auf beiden Nasenseiten beim „in-concert“ Typ vermuten Mlynski et al. einen Kompensationsmechanismus der Nase. Steigt bei leichter körperlicher Belastung der Bedarf der Luftzufuhr, so werde dies durch den Übergang beider Nasenseiten in die Arbeitsphase ermöglicht [19]. Zudem wurde beobachtet, dass dieser Zyklustyp bei Kindern gehäuft vorkommt [19]. Das lässt vermuten, dass es im Wachstum zu einer Veränderung des nasalen Schwellungsverhaltens und damit auch zu einer Verschiebung des Aufgabenbereiches der Nase kommt. Studien bei Kindern sind jedoch bisher sehr selten [3].

Ursächlich für den „einseitigen“ Nasenzyklus, also den Nachweis von Fluktuationen des nasalen Luftflusses nur auf einer Seite, könnte ein strukturelle Enge der Gegenseite sein [19]. Wir konnten diesen Zyklustyp jedoch auch bei einem Teil unserer nasengesunden Probanden ohne Deviation der äußeren oder inneren Nase nachweisen. Wie Abb. 5 zeigt, kommt dieser Typ auch neben anderen Zyklustypen innerhalb der 24-stündigen Messung bei einigen Probanden vor.

Das komplette Fehlen einer messbaren Schwankung des nasalen Luftflusses erklärten sich Mlynski et al. durch eine iatrogene Intervention, also z. B. eine komplette Resektion der nasalen Schwellkörper [19]. Bei unseren gesunden, nicht operierten Patienten konnten wir den „non-cycle“ Typ nachweisen, wenn auch selten.

Den in der Literatur erwähnten [1, 25] „irreguläreren Typ“, ohne erkennbares Muster oder ein konstantes Atemvolumen, konnten wir nicht nachweisen.

Ziel dieser Arbeit war es zunächst, die beobachteten Schwellungsmuster zu beschreiben. Eine nähere Korrelation der Daten sowohl mit der Aktivität als auch mit dem Alter der Probanden steht noch aus. In unserer Auswertung waren die Zyklustypen jeweils gut voneinander abgrenzbar, und ein Schwellungsmuster lag jeweils über mehrere Stunden vor. Eine nähere Evaluation der Kurven, z. B. zur Definition der Mindestdauer eines Zyklustypen oder zur Festlegung eines relativen Verhältnisses zwischen den Flow-Kurven beider Nasenseiten, kann ebenfalls helfen, die Erforschung des Nasenzyklus zu vereinheitlichen.

### Die Langzeitrhinometrie

Bisher stützte sich die Erforschung des Nasenzyklus hauptsächlich auf einzeitige Messmethoden. Eine der aktuelleren Untersuchungen ist die Studie der Arbeitsgruppe um Pendolino aus dem Jahr 2018 [24]. Hier wurden die Veränderungen des nasalen Luftflusses seitengetrennt mit der PNIF („peak nasal inspiratory flow“) und der Rhinomanometrie nachgewiesen. Bei der Hälfte der Probanden wurde so ein reziprokes, bei der anderen Hälfte ein simultanes Schwellungsmuster nachgewiesen. Ein Proband wies keine Fluktuation auf, dies wurde als „non-cycle nose“ gewertet [24]. Auch hier ist aus unserer Sicht die Beurteilbarkeit der Ergebnisse begrenzt, da eine Flusskurve aus 4 Einzelmessungen über 8 h erstellt wurde. Zudem wurde den Probanden vor der Messung eine Zeit zur „Akklimatisierung“ eingeräumt, sodass durch das Studiendesign bereits die Aussage auf den Zustand der „leichten körperlichen Aktivität“ beschränkt ist.

Einzeitige Messmethoden bedeuten einen hohen Aufwand für Untersucher und Proband und machen eine Unterbrechung von Aktivitäten oder Schlafphasen erforderlich. Die LRM ist eine nichtinvasive Messmethode, die bis auf die Kalibrierung zu Beginn keine weitere Vorbereitung benötigt. Im klinischen Alltag und in dieser Studie erwies sie sich als wenig störanfällig und wurde von den meisten Probanden gut angenommen. So ermöglicht die LRM eine Abbildung des nasalen Schwellungsverhaltens sowohl bei sportlichen Aktivitäten, als auch im Schlaf. Die LRM kann Probanden/Patienten ähnlich wie ein Gerät zur ambulanten Polygraphie ausgehändigt werden.

Die Erforschung des Nasenzyklus konzentrierte sich bisher hauptsächlich auf die Nachweisbarkeit von streng wechselseitigem An- und Abschwellen der nasalen Mukosa. Je nach Methode, Messdauer und Definition des Nasenzyklus variierten die Angaben stark zwischen 13 % [8] und 100 % [29]. Bis zum heutigen Zeitpunkt (Juni 2021) finden sich erst wenige Studien über den Nasenzyklus mit der LRM. Ohki et al. führten 2005 jeweils an 20 Probanden zwischen 24 und 77 Jahren Untersuchungen mit einem tragbaren Rhinoflowmeter (Rhinometrics A/S; Smørum, Dänemark) durch und konnten innerhalb des 12-stündigen Messzeitraumes am Tag bei 14 Probanden einen klassischen Nasenzyklus nachweisen [22]. Mlynski et al. führten 2005 erstmals Untersuchungen mit der LRM der Firma Happersberger Otopront durch. Der „klassische Nasenzyklus“ mit Phasen zwischen 90 min und 10 h konnte dargestellt werden [9]. Durch die aufwendigen Einzelmessungen wurde zuvor meist eine Untersuchungszeit von 8 h nicht überschritten, was einen Nasenzyklus mit sehr langen Phasen ggf. nicht abdeckte. Im Jahr 2012 untersuchten Braun et al. mit der LRM der Happersberger Otopront GmbH die Effekte von Oxymetazolin Nasenspray auf den Nasenzyklus [2], 2014 Rohrmeier et al. untersuchten den Nasenzyklus über 24 h in Relation zur Körperposition [26]. Ein klassischer Nasenzyklus konnte in letztgenannter Studie bei 50 % der 20 Probanden im Wachzustand und bei 75 % im Schlaf detektiert werden. Die Phasen waren jeweils während des Schlafes signifikant verlängert [26]. Auch in unserer Untersuchung scheint sich die Verteilung der Nasenzyklustypen zu unterschiedlichen Tageszeiten zu unterscheiden. Die Literatur deutet darauf hin, dass physiologische und pathologische Faktoren, wie Körperlage, Schlaf, körperliche Belastung und Allergien, das Schwellungsverhalten beeinflussen [23, 25].

## Ausblick

Die Ergebnisse der LRM demonstrieren ein komplexes Bild des Nasenzyklus. In den meisten Fällen zeigen die Probanden mehrere der oben genannten Zyklustypen im Verlauf der 24-h-Messung und damit ein gemischtes Bild. Wir vermuten daher, dass nicht jedem Menschen ein bestimmter Zyklustyp zugeordnet werden kann, sondern dass das Schwellungsverhalten der Nase wesentlich von innerer Rhythmik und äußeren Einflüssen abhängt. Mit der LRM liegt nun aus unserer Sicht eine einfach zu handhabende Methode vor, die in Studien zur Erforschung verschiedener Einflüsse auf die zugrundeliegende Physiologie des Nasenzyklus ein besseres Gesamtbild ermöglicht.

Im Rahmen der Grundlagenforschung kann so der Zusammenhang zwischen dem Nasenzyklus und Faktoren, wie dem Alter und körperlicher Aktivität sowie Schlaf, mittels der LRM über 24 h unter Alltagsbedingungen genauer untersucht werden. Auch die Auswirkungen verschiedener rhinologischer Krankheitsbilder, wie anatomische Varianten und Formstörungen oder nasale Polypen, können so besser nachvollzogen werden. Zudem wäre es interessant, die Messungen bei einzelnen Probanden über mehrere Tage durchzuführen, um äußere Einflüsse besser zu verstehen. Dies könnte auch helfen, temporäre Beschwerden der Patienten, die sich im klinischen Alltag nicht immer nachvollziehen lassen, besser einzuschätzen und so die Operationsplanung zu verbessern. Medizinische Indikationen der LRM wären z. B. Indikationsprüfung einer Nasenmuschelplastik, unklare nächtliche nasale Obstruktion mit/ohne anatomische Korrelate, obstruktive Schlafapnoe, allergische Rhinitis (z. B. Hausstaubmilben, Berufsallergien), das „empty nose syndrome“ oder komplexe Revisionseingriffe an der Nase.

## Fazit für die Praxis

- Die Physiologie und die Funktion des Schwellungsverhaltens der nasalen Mukosa sind bis heute nicht abschließend verstanden, was die Patientenversorgung beeinträchtigen kann.
- Die Befunde der Langzeitrhinometrie (LRM) über 24 h lassen vermuten, dass das Schwellungsverhalten wesentlich von innerer Rhythmik und äußeren Einflüssen abhängt.
- Wir schlagen die folgenden Zyklustypen vor: „klassischer“ Typ mit reziprokem Wechsel aus An- und Abschwellen der Schleimhaut beider Nasenseiten, „in-concert“ Typ mit simultanem Anstieg und Abfall des Luftstromes auf beiden Nasenseiten, „einseitiger“ Typ mit An- und Abschwellen der Schleimhaut nur auf einer Nasenseite, „non-cycle“ Typ ohne jegliche Darstellbarkeit einer Veränderung des Luftstromes auf beiden Nasenseiten, und, da im Verlauf der 24-h-Messung mehrere Formen auftreten können, den „gemischten“ Nasenzyklus.
- Die LRM ermöglicht die Erforschung des Schwellungsverhaltens unter Alltagsbedingungen.
- Sie stellt ein wertvolles Messinstrument zur Beurteilung der Symptomatik und zur Operationsplanung dar.
